# Cytotoxicity of fractured quartz on THP-1 human macrophages: role of the membranolytic activity of quartz and phagolysosome destabilization

**DOI:** 10.1007/s00204-020-02819-x

**Published:** 2020-06-26

**Authors:** Riccardo Leinardi, Cristina Pavan, Harita Yedavally, Maura Tomatis, Anna Salvati, Francesco Turci

**Affiliations:** 1 “G. Scansetti” Interdepartmental Center for Studies On Asbestos and Other Toxic Particulates, Department of Chemistry, University of Torino, Via P. Giuria 7, 10125 Turin, Italy; 2 Louvain Centre for Toxicology and Applied Pharmacology (LTAP), Université Catholique de Louvain, Avenue Hippocrate 57, 1200 Brussels, Belgium; 3 Department of Nanomedicine & Drug Targeting, Groningen Research Institute of Pharmacy (GRIP), University of Groningen, Antonius Deusinglaan 1, Groningen, 9713 AV The Netherlands

**Keywords:** Quartz cytotoxicity, Quartz surface, Macrophages, Membrane, Phagolysosome

## Abstract

The pathogenicity of quartz involves lysosomal alteration in alveolar macrophages. This event triggers the inflammatory cascade that may lead to quartz-induced silicosis and eventually lung cancer. Experiments with synthetic quartz crystals recently showed that quartz dust is cytotoxic only when the atomic order of the crystal surfaces is upset by fracturing. Cytotoxicity was not observed when quartz had as-grown, unfractured surfaces. These findings raised questions on the potential impact of quartz surfaces on the phagolysosomal membrane upon internalization of the particles by macrophages. To gain insights on the surface-induced cytotoxicity of quartz, as-grown and fractured quartz particles in respirable size differing only in surface properties related to fracturing were prepared and physico-chemically characterized. Synthetic quartz particles were compared to a well-known toxic commercial quartz dust. Membranolysis was assessed on red blood cells, and quartz uptake, cell viability and effects on lysosomes were assessed on human PMA-differentiated THP-1 macrophages, upon exposing cells to increasing concentrations of quartz particles (10–250 µg/ml). All quartz samples were internalized, but only fractured quartz elicited cytotoxicity and phagolysosomal alterations. These effects were blunted when uptake was suppressed by incubating macrophages with particles at 4 °C. Membranolysis, but not cytotoxicity, was quenched when fractured quartz was incubated with cells in protein-supplemented medium. We propose that, upon internalization, the phagolysosome environment rapidly removes serum proteins from the quartz surface, restoring quartz membranolytic activity in the phagolysosomes. Our findings indicate that the cytotoxic activity of fractured quartz is elicited by promoting phagolysosomal membrane alteration.

## Introduction

Exposure to crystalline silica dusts, in particular quartz, induces severe toxic effects in humans (American Thoracic Society 1997; Parks et al. 1999; Leung et al. 2009; IARC 2012). It is also known that a cluster of dust properties, including particle size (Wiessner et al. 1989; Fenoglio et al. 2000), the capacity to induce free radicals (Vallyathan et al. 1988; Dalal et al. 1990; Schins et al. 2002), and the degree of hydrophilicity, related to the distribution of siloxanes and silanol families on the particle surface (Bolis et al. 1991; Hemenway et al. 1994), give a contribution to the adverse effects of quartz in vitro and in vivo. The various adverse cellular effects reported in the literature are related to particle-membrane interactions (Warheit et al. 2007), and to the activation of lung cells (Shen et al. 2001; Gilberti et al. 2008; Giordano et al. 2010). Recently, a revisited mechanism of toxicity for quartz particles in the alveolar space has been proposed (Pavan and Fubini 2017). According to this model, inhaled particles are recognized and internalized by alveolar macrophages to be cleared out from the lungs (Hamilton et al. 2008). The deposition of non-cleared particles onto the alveolar epithelium causes the recruitment and the activation of new macrophages and neutrophils, in a self-sustained detrimental process which gives rise to persistent inflammation, as long as the particles remain in the alveolar space**.**

The alveolar macrophages (AM) are among the first cells of the body to have significant contact with inhaled particles (Hamilton et al. 2008; Hiraiwa and Van Eeden 2013). Because of the active phagocytosis of AM, quartz particles mainly interact with the macrophage phagolysosomal membrane (Joshi et al. 2015). It is, in fact, known that most particles accumulate at lysosomal level following uptake, and because of this, the particle impact on the phagolysosomes is a key parameter to be investigated (Rejman et al. 2004; Nel et al. 2009; Salvati et al. 2011; Wang et al. 2013; Sabella et al. 2014). Upon quartz phagocytosis, lysosome membrane permeabilization (LMP) may occur and, the lysosomal content, such as cathepsins B and S (Wang et al. 2013; Hughes et al. 2015), may leak into the cytosol. As recently evidenced, this event can trigger the NALP3 inflammasome machinery (Hughes et al. 2015; Sayan and Mossman 2015), which in turn causes activation of the proteolytic enzyme caspase-1 and the release of active pro-inflammatory cytokines (i.e. IL-1β and IL-18) (Pavan et al. 2014; Rabolli et al. 2015; Hindman and Ma 2019). The interaction of quartz particles with the phagolysosomal membrane, a crucial step according to the model of quartz-induced inflammogenicity, was recently proposed as the molecular initiating event (MIE) of the quartz adverse outcome pathway (AOP) towards persistent inflammation and silicosis (Pavan and Fubini 2017). In search for the molecular determinant of this interaction, a considerable number of works have been published in the last few years (Pavan et al. 2013, 2014, 2017; Turci et al. 2016; Joshi et al. 2017). Traditionally, a well-established biomembrane model is represented by the red blood cell (Nolan et al. 1981), and hemolysis is largely used to probe the interaction of amorphous and crystalline silica particles with biomembranes (Gerashchenko et al. 2002; Zhao et al. 2011; Pavan et al. 2013). Recently, we have demonstrated that synthetic quartz crystals are hemolytic only upon mechanical fracturing, a procedure that upsets the expected long-range order of quartz non-radical surface moieties (such as silanols, silanolates, siloxanes) (Turci et al. 2016). In previous studies, several approaches were developed to modulate cellular responses to quartz by modifying its surface in many different ways (Daniel et al. 1995; Duffin et al. 2001; Fubini and Hubbard 2003). However, a clear connection between the surface states and features of different quartz particles and their impact on cellular membranes, and more particularly the phagolysosomal membrane, still needs to be established.

Here, we investigated the impact of quartz particles on human macrophages, specifically analyzing whether quartz can induce alterations of the phagolysosome membrane. To this aim, we compared the effect on cell viability and impact at the lysosomal level of three surface-differentiated quartz specimens, namely: an ad-hoc synthesized quartz crystal in respirable size (Pastero et al. 2016), exposing as-grown, unfractured, regular faces; a synthetic quartz mechanically fractured down to a size similar to the as-grown quartz; and a cytotoxic commercial quartz dust used in several toxicological works, as a positive control (Allison 1966; Castranova 2004; Lison et al. 2009; Pfau et al. 2012). Human red blood cells were used as a model of the cellular membrane (not capable of internalization) for assessing quartz membranolytic activity and for evidencing the effect of protein adsorption on the membranolytic activity of fractured quartz. PMA-differentiated THP-1 cells were selected as a common model for human alveolar macrophages (Theus et al. 2004; Wottrich et al. 2004; Kletting et al. 2018). Particle uptake was monitored by transmission electron microscopy (TEM) and a combination of cell viability and flow cytometry-based assays was used to determine alterations induced at lysosomal level. This work provides novel and fundamental insights on the mechanisms of the pathogenicity of quartz, clarifying the nature of the molecular initiating event resulting from the interaction of quartz crystals with the phagolysosomes, following particle internalization by macrophages.

## Materials and methods

### Crystalline silica samples

Two synthetic α-quartz samples with as-grown (gQ) or fractured (gQ-f) faces were used in this study. A commercial fractured α-quartz dust, largely used in studies of experimental silicosis and lung cancer (Min-U-Sil 5 quartz, US Silica Co., Berkeley Springs, WV, USA, lot number 15062696, in this work named cQ-f), was used as positive control.

Synthetic quartz crystals with as-grown regular faces (gQ) were obtained following the procedure developed by Pastero and coworkers (Pastero et al. 2016). Briefly, a 25% w/w Na-metasilicate (Na-MTS) solution was polymerized into gel by the addition of 1 m HNO_3_. The gel was stabilized at pH ≈ 8. Growth runs were performed in polytetrafluoroethylene (PTFE) liner steel autoclaves at 210 °C, for 168 h. The finest fraction (< 30 μm), obtained through 30 min of sieving in 100 and 30 μm sieves, was used in all the experiments. To obtain crystals with fractured faces (gQ-f), a further synthesis was carried out, where the Na-MTS solution was polymerized by bubbling CO_2_ until gel formation, at pH ca. 8. By growing quartz with CO_2_ as polymerizing agent, we synthesized large crystals that could be conveniently ground in a ball mill, obtaining fractured crystals with size and specific surface area well comparable to gQ crystals. The largest fraction (> 30 μm), obtained as above, was mechanically fractured by milling in a MM 200 mixer mill (Retsch, Haan, Germany) in agate jars (27 Hz, two spheres, 500 mg of dust/jar) for 6 h, to induce surface alterations. Thus, following this procedure, we were able to obtain as-grown (gQ) and fractured (gQ-f) crystals, that shared very similar physicochemical properties (particle size distribution, chemical composition, surface area, surface charge at physiological pH) and only differed in the occurrence of surface structures induced by fracturing.

### Particle size distribution

Particle size distribution was determined by Flow Particles Image Analyzer (FPIA), using a Sysmex FPIA 3000 particle size and shape analyser (Malvern Panalytical, Malvern, UK) and by dynamic light scattering (DLS), using a Zetasizer Nano ZS (Malvern Instruments, Malvern, UK). For FPIA analysis, a sample dispersion of 0.5 mg/ml in Milli-Q water was prepared and probe sonicated on ice for 3 min at 30% amplitude, power 25 W, energy delivered into the sample 0.450 kJ/ml, using a Sonopuls HD3100 homogeniser (Bandelin, Berlin, Germany). For DLS analysis, a sample dispersion in Roswell Park Memorial Institute (RPMI) 1640 medium (0.5 mg/ml), with the addition of 10% foetal bovine serum (FBS), was prepared and sonicated as described.

### Surface area determination

Specific surface area (SSA) was evaluated by the Brunauer–Emmett–Teller (BET) method, based on Kr adsorption. Quartz samples were firstly outgassed for 2 h, at 150 °C. The analysis was then performed at − 196 °C using an ASAP 2020 physisorption analyser (Micromeritics, Norcross, USA).

### ζ-Potential

The ζ potential of the quartz samples was evaluated by means of electrophoretic light scattering (ELS) with a Zetasizer Nano–ZS (Malvern-Panalytical, Malvern, UK). In this technique, the velocity of a particle in an oscillating electric field, which is proportional to its ζ potential, is measured by light scattering. The ζ potential was measured after suspending quartz (0.5 mg/ml) in serum-free RPMI medium (s.f. RPMI) or RPMI + 10% FBS (complete RPMI medium, cRPMI), to evaluate the surface charge at exposure conditions (pH ca. 8) and the modulation given by serum proteins. Investigation of zeta potential at lysosomal pH (pH 4.5) was measured suspending quartz particles (0.5 mg/ml) in 0.01 M NaCl, and adjusting the pH of the suspension to the experimental value with 0.1 M HCl or 0.1 M NaOH.

### Cell culture and differentiation

RPMI medium, FBS and Dulbecco’s phosphate-buffered saline (DPBS) were purchased from Gibco (Thermo Fischer Scientific, Waltham, MA, USA). Experiments were performed on human THP-1, a human monocyte-like cell line derived from a patient with leukaemia, obtained from ATCC bank (ATCC#TIB-202, RRID: CVCL_0006) (Tsuchiya et al. 1980). Cells suspensions were cultured in RPMI 1640 supplemented with 10% FBS (cRPMI), at 37 °C and 5% CO_2_. After seeding (200,000 or 100,000 cells/well in a Cellstar 24-well plate for LysoTracker staining and propidium iodide (PI) staining, respectively, or 50,000 cells/well in a Cellstar transparent 96-well plate for MTT assay), cells were differentiated into macrophages by incubation with 100 nM phorbol 12-myristate 13-acetate (PMA) in cell culture medium for 48 h at 37 °C and 5% CO_2_ (Tsuchiya et al. 1982). Transparent 24-well and 96-well plates were purchased from Greiner Bio-One (Greiner Bio-One, Kremsmunster, Austria).

### TEM imaging

Particle uptake by THP-1 macrophages was investigated by means of transmission electron microscopy on cross sections of fixed cells, upon 24 h incubation with 50 µg/ml of synthetic or commercial quartz samples. After exposure, the cells were fixed with 0.2% glutaraldehyde and 2% paraformaldehyde (PFA) in 0.1 M sodium cacodylate buffer (pH 7.4) for 1 h. Then, cells were rinsed twice for 5 min in 0.1 M cacodylate buffer at room temperature followed by post-fixation in 1% osmium tetroxide/1.5% potassium ferrocyanide in 0.1 M sodium cacodylate at 4 °C for 30 min. The cells were then washed with Milli-Q water, dehydrated through serial incubation in a graded ethanol series (30, 50, 70 and 100%), and lastly embedded in EPON resin and polymerized at 37 °C for 16 h followed by 58 °C for 24 h. Given the size of the particles and the fact that they are hard to cut with a standard diamond knife, sections were cut at a thickness of 200 nm instead of the standard 80 nm using an UC7 ultramicrotome (Leica, Vienna, Austria). This allowed to partially reduce the presence of holes in the section, corresponding to areas were particles accumulate and that cannot be sectioned, while still being able to determine whether particle uptake was present. Sections were then contrasted using 5% uranyl acetate for 20 min, followed by Reynolds lead citrate for 2 min. Images were recorded with a CM100 Biotwin transmission electron microscope (FEI, Eindhoven, the Netherlands) operated at 80 kV using a Morada digital camera.

### MTT assay

Cell metabolic activity was assessed through a 3-(4,5-dimethylthiazol-2-yl)-2,5-diphenyltetrazolium bromide (MTT) assay (Gerlier and Thomasset 1986). After differentiation, THP-1 macrophages, previously plated at a density of 50,000 cells/well in a transparent 96-well plate, were exposed for 24 h to quartz particles at increasing concentrations (10, 25, 50, 100, 250 μg/ml) in complete medium (cRPMI). Before the assay, each well was washed with Dulbecco’s Phosphate-Buffered Saline (DPBS) to remove the extracellular particles and eventual cell debris and treated with MTT (0.5 mg/ml) in cRPMI for 20 min at 37 °C. Then, the MTT reagent was discarded and dimethyl sulfoxide (DMSO) was added to each well (200 µl) to solubilise formazan crystals. Each well was pipetted again to mix, and absorbance at 550 nm was measured using a THERMOmax microplate reader (Molecular Devices, San Josè, CA, USA). All values were normalized to the results obtained in untreated cells.

MTT assay was also carried out after 4 h of exposure at 4 °C, to block active cellular processes (Dunn et al. 1980; Sharma et al. 2010). Plates with cells were pre-incubated at 4 °C for 30 min, just before the exposure to quartz particles (25, 50, 100, 250 μg/ml) in cRPMI. After 4 h of incubation, wells were washed three times with cRPMI (100 μl), and further incubated in fresh medium without particles for 20 h at 37 °C and 5% CO_2_. The same protocol was applied for 4-h exposure at 37 °C. To assess protein modulation on quartz cytotoxicity, the MTT assay was carried out as previously explained, exposing PMA-differentiated THP-1 cells (50,000 cells/well) for 4 or 24 h to quartz samples (100 μg/ml), in s.f. RPMI or cRPMI supplemented with 10% FBS. The analysis was carried out as described above. MTT results are the mean of four biological replicates of one representative experiment out of three.

### Lyso Tracker staining and flow cytometry (FACS)

The impact on the lysosomes after interaction and internalization of particles was investigated by staining cells with Lyso Tracker Red DND-99 (Thermo Fisher Scientific, Waltham, MA, USA), a fluorescent acidotropic probe for labelling and tracking acidic organelles in live cells. Increase in Lyso Tracker intensity from the basal condition can be due to an increase in lysosomal volume, lysosomal number, or lysosomal acidity. THP-1 cells differentiated into macrophages were exposed to particle dispersions (25, 50, 100 μg/ml) prepared by diluting a stock dispersion (1 mg/ml) in cRPMI. Cells were grown on transparent 24-well plates (200,000 cells/well), differentiated for 48 h as described above, and then exposed to particles. After 24 h exposure, at 37 °C and 5% CO_2_, wells were washed (1 × 500 μl with the complete medium) to remove quartz particles, then cells were incubated 15 min at 37 °C, 5% CO_2_, with a solution (25 nmol, 500 μl) of Lyso Tracker Red DND-99 (Thermo Fisher Scientific, Waltham, MA, USA), in cRPMI. Then, the dye solution was discarded and wells were washed again with cRPMI (1 × 500 μl) and DPBS (2 × 500 μl). Cells were detached with 0.05% trypsin/EDTA (300 μl) at 37 °C for 5 min, followed by treatment with EDTA solution (5 mmol) in PBS (pH 7.4), at 37 °C. Then, cells were harvested in FACS tubes and pelleted by centrifugation at 250 g for 3 min. Cells were resuspended in DPBS (100 μl) and analysed using a CytoFlex flow cytometer (Beckman-Coulter, Brea, CA, USA). LysoTracker Red fluorescence intensity was analyzed in the FL3 channel. Quantitative analysis of flow cytometry data was carried out using the FlowJo software (TreeStar Inc., Ashland, OR, USA). Gates were set to discriminate cell debris and cell doublets from the analysis, according to their forward and side scattering. 20,000 cells were acquired, unless specified in the case of samples for which strong cytotoxicity was detected (in these cases a variable number of cells, ranging from 20,000 to at least ca. 7000 was acquired, as specified in figure captions). The same experiment was performed after 4 h exposure at 4 and 37 °C, for cells exposed to 50 and 100 µg/ ml of particles. The exposure at 4 °C was preceded by a 30-min pre-incubation step of cells, at the same temperature.

The shown Lyso Tracker Red data are the result of three biological replicates of one representative experiment out of three.

### Propidium iodide (PI) assay

THP-1 cells differentiated into macrophages were seeded on transparent 24-well plates (100,000 cells/well) and, 48 h after seeding, cells were exposed to particles (final concentration: 100 μg/ml) in serum-free (- FBS) or complete RPMI (+ FBS). After 4 h exposure at 37 °C and 5% CO_2_, quartz dispersions were discarded and wells were washed with cRPMI (1 × 500 μl). Then, cells were harvested as described, collected into FACS tubes and stained with a PI solution (5 μg/ml) at room temperature. After incubation with the dye (15 min, 37° C), samples were measured as described above. A variable number of cells, ranging from 12,000 to ca. 7000, were acquired for each sample (as specified in the figure caption). PI assay data result from one representative experiment out of three, using four replicates. Data were analyzed using the FlowJo software (Tree Star Inc., Ashland, OR, USA).

### Hemolysis assay

The hemolytic activity of quartz particles refers to a method described by Lu & coworkers (Lu et al. 2009), with minor modifications given by Pavan & coworkers (Pavan et al. 2013). Red blood cells (RBCs) were separated from fresh human blood of healthy volunteer donors, not receiving any pharmacological treatment. To assess the effect of FBS proteins on quartz hemolytic activity, particles were incubated with increasing concentrations of FBS (0.03%, 0.06%, 0.3%) in the exposure medium (DPBS). Briefly, quartz suspensions (75 μl/well,) at a concentration of 0.37 mg/ml were dispensed in a transparent 96-well Cellstar microplate (Greiner Bio-One, Kremsmunster, Austria), and FBS (75 μl/well) was added, to each well. After 30 min of incubation at room temperature (25 °C), the red blood cell suspension (75 μl/well) was added, and the plate, gently shaken on an orbital plate shaker, was incubated for 30 min at 25 °C.

The plate was centrifuged for 5 min at 1200 RPM, using a Centrifuge Rotina 420R centrifuge (Hettic Instruments, Beverly, MA, USA). The supernatant (75 μl) was removed and transferred to a clean transparent microplate. The amount of haemoglobin released into the supernatant was spectrophotometrically determined at a wavelength of 540 nm with an Infinite 200 UV/Vis spectrophotometer (Tecan, Grödig, Austria). Absorbance values were converted into percentages of haemolysis according to the formula (Eq. 1):

1 $$\% {\text{Hemolysis}} = \frac{{\left( {{\text{Sample OD}} - {\text{Negative Control Average OD}}} \right)}}{{\left( {{\text{Positive Control Average OD}} - {\text{Negative Control Average OD}}} \right)}} \times 100$$

where OD is the optical density or absorbance.

DPBS and 0.1% Triton-X100 were used as negative and positive controls, respectively. Released hemoglobyn did not adsorb on quartz samples (data not shown).

### Statistical analysis

Statistical analysis was performed using Systat 10 (Systat Software Inc., San Jose, CA, USA), and carried out by one-way analysis of variance (ANOVA) followed by Tukey’s post hoc test, as appropriate. Differences with *p* value < 0.05 were considered statistically significant.

## Results

### As-grown and fractured crystals share similar physico-chemical properties

To study the impact of quartz particles on phagolysosome membrane, two synthetic quartz samples, with as-grown regular (gQ) or fractured (gQ-f) surfaces were prepared, from a similar synthesis (Pastero et al. 2016). A commercial quartz dust (cQ-f) was used as a positive control, due to its known ability to induce cytotoxicity, increase in lysosomal permeability, and inflammogenic activity (Thibodeau et al. 2004; Dostert et al. 2008; Hornung et al. 2008; Sellamuthu 2011). Overall, physico-chemical characterization results showed that samples share both particle size distribution and, albeit to a lesser extent, surface charge, under the test conditions (Table 1).

**Table 1:** Main physico-chemical properties of the investigated samples. Synthetic samples (gQ, as grown; gQ-f, fractured) were characterized and compared to a commercial quartz dust (cQ-f)

Sample	Origin	Surface state	FPIA Size^a^ (μm ± s.d.)	DLS diameter^b^ (μm ± s.d.) (PDI)	SSA^c^ (m^2/^g)	ζ-potential (mV ± s.d.)		
						s.f. RPMI^d^	cRPMI^e^	0.01 NaCl^f^
gQ	Synthetic	As-grown	1.3 ± 2.3	0.89 ± 0.23 (0.507)	5.8	ca. 0	− 11 ± 1	− 45 ± 2
gQ-f	Synthetic	Fractured	1.2 ± 0.7	1.55 ± 0.18 (0.485)	4.5	ca. 0	− 12 ± 1	− 50 ± 1
cQ-f	Commercial	Fractured	1.0 ± 1.2	1.74 ± 0.20 (0.295)	4.3	ca. 0	− 10 ± 1	− 59 ± 2

Size distribution of crystals was evaluated in Milli-Q water by flow particle image analyser and in cRPMI by dynamic light scattering. Specific surface area was evaluated via BET method, by Kr adsorption. The ζ-potential was evaluated in the different medium at specific pH, by electronic light scattering

^a^Measured by flow particle image analyser (FPIA) in ultrapure H_2_O

^b^Measured by dynamic light scattering (DLS) in + RPMI + 10% FBS, the polydispersity index (PDI) is reported in brackets

^c^Measured by Kr-BET method

^d^Measured by electrophoretic light scattering (ELS) in serum free RPMI (pH ca. 8)

^e^Measured by electrophoretic light scattering (ELS) in RPMI + 10% FBS (pH ca. 8)

^f^Measured by electrophoretic light scattering (ELS) in 0.01 m NaCl (pH 4.5)

**Fig. 1:**
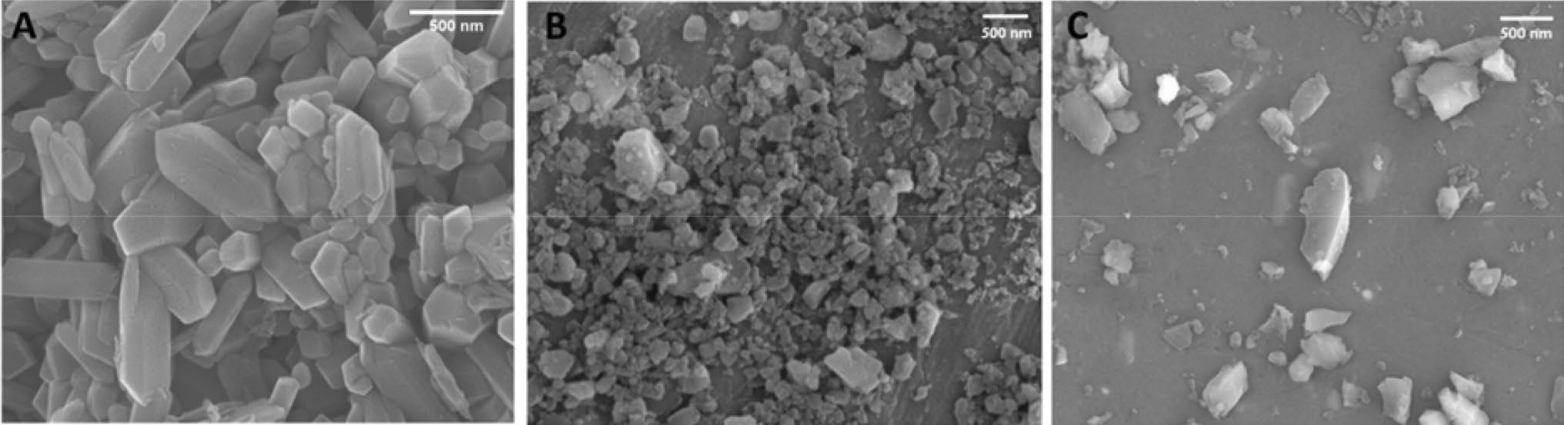
FE-SEM imaging of gQ, gQ-f and cQ-f quartz samples. **a** Synthetic regular quartz crystals (gQ) show as-grown, flat and smooth surfaces; fractured quartz crystals (**b**, **c**) (gQ-f and cQ-f, respectively) exhibit irregular surfaces and the typical fractures due to mechanical fracturing

Dimensional characterization showed that the samples had a respirable micrometric size. Field-emission scanning electron microscopy (FE-SEM) analysis confirmed that as-grown gQ crystals had a well-organized morphology, with flat and smooth surfaces. Fractured gQ-f and cQ-f exhibited a morphology typical of commercial quartz dust, (Fig. 1), as previously evidenced (Murashov and Demchuk 2005). Specific surface area (SSA, Kr-BET) and particle size distribution, evaluated by dynamic light scattering (DLS) and flow particle image analysis (FPIA), of the synthetic samples, were comparable with the positive control quartz (Table 1). The SSA of gQ-f was slightly lower than that of gQ, because the latter contains a slightly larger fraction of fine particles, as evidenced by the DLS analysis.

The zeta potential of particles (ELS, measured in the various medium at specific pH), here used to describe the average acidity of surface silanols (Pavan et al. 2017), acidic moieties with a potential for H-bonding, showed no significant differences among the set of samples, for the same medium. As expected, the zeta potential of particles dispersed in saline (0.01 M NaCl) at pH 4.5, which represents the lysosomal pH, was strongly negative (<  − 40 mV). Only small differences among zeta potential, namely gQ being less negatively charged than gQ-f and cQ-f, were detected. These differences were assigned to the different average acidity of the surface silanols exposed at the quartz surface. Silanols (≡Si–OH) are known to behave in aqueous media as weak monoprotic Brønsted acid, exhibiting a dissociation constant that is dependent on the silanol structural arrangement with respect to neighbouring silanols (Hiemstra et al. 1989; Bolis et al. 1997; Pavan et al. 2017). Silanols at silica surface can thus be described as families of silanols, sharing similar acidic properties that impart as a whole a specific surface charge (Pavan et al., under review/unpublished work). Thus, the slight difference in surface charge between gQ and gQ-f crystals suggests the occurrence of different silanol families with different chemical properties in the quartz particles, that were induced by fracturing and confirms previous findings (Turci et al. 2016). Such a difference was not observed when particles were dispersed in serum-free RPMI where, consistent with the much higher ionic strength of RPMI, all samples exhibited a zeta potential close to the point of zero charge (pzc). The measure of the zeta potential was also used to evaluate the adsorption of serum proteins on the quartz surface (Turci et al. 2010). Upon dispersion of particles in complete RPMI, all quartz samples exhibited a negative zeta potential (ca. − 10 mV), compatible with the adsorption of serum proteins on the particle surface and the formation of the protein corona.

### As-grown and fractured crystals are both internalized by human macrophages

**Fig. 2:**
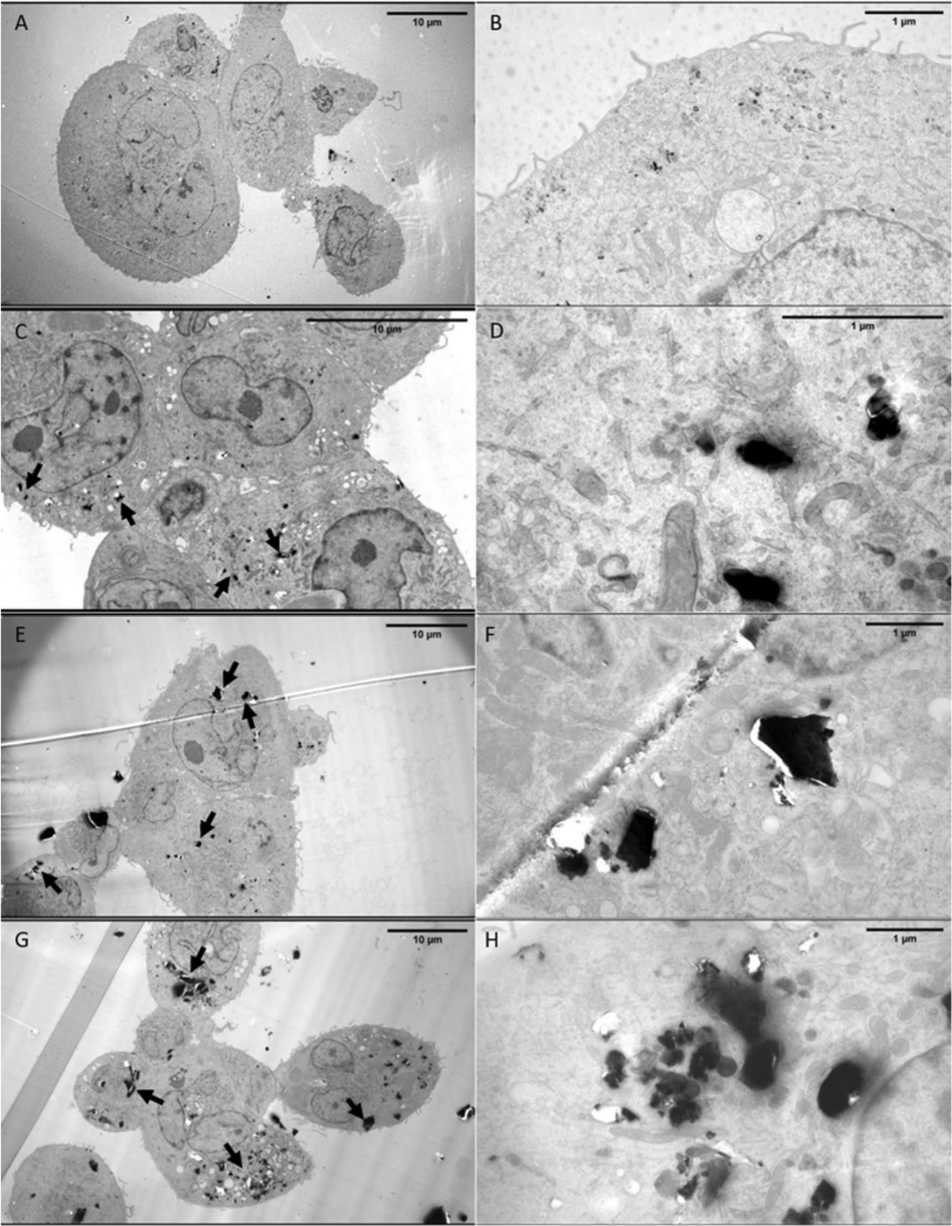
Representative images by transmission electron microscopy (TEM) of differentiated THP-1 human macrophages exposed to quartz particles. Cells were exposed to synthetic (**c**, **d**) as-grown (gQ) and (**e**, **f)** fractured (gQ-f) quartz particles (50 µg/ml) for 24 h. **a**, **b**) show equivalent images of untreated cells, and (**g, h**) cells exposed to a positive control (cQ-f). Electron microscopy confirmed that all types of particles (black arrows) were internalized by macrophages. Scale bars: 10 μm (**a**, **c**, **e**, **g**), and 1 µm (**b**, **d**, **f**, **h**)

The uptake and cytotoxicity of the different quartz particles were evaluated on PMA-differentiated THP-1 human monocytes, here chosen as a common model for human alveolar macrophages (Theus et al. 2004; Wottrich et al. 2004; Kletting et al. 2018). Transmission electron microscopy (TEM) of thin cross-sections of quartz-incubated and fixated cells indicated that all the samples were internalized by the macrophages (Fig. 2), confirming the previous finding in murine macrophages (Turci et al. 2016). To take into account the hardness and particle dimension of quartz, sections were prepared with a larger thickness than conventional bio-TEM sections. No evidence of particles absorbed on the outer cell membrane was found. Inside the cells, both single particles and micrometric aggregates could be visualized. Due to the larger thickness of the sections and also the high density of the particles with respect to the cellular background, it was not clear whether the internalized particles were enclosed in vesicles, as expected following some form of endocytosis (Fig. 2d, f, h show details at higher magnification). In addition, section thickness impaired the observation of lysosomes, which usually are easy to recognize as darker organelles inside cells by TEM.

### Fractured quartz reduces cell viability and induces phagolysosomal alterations

**Fig. 3:**
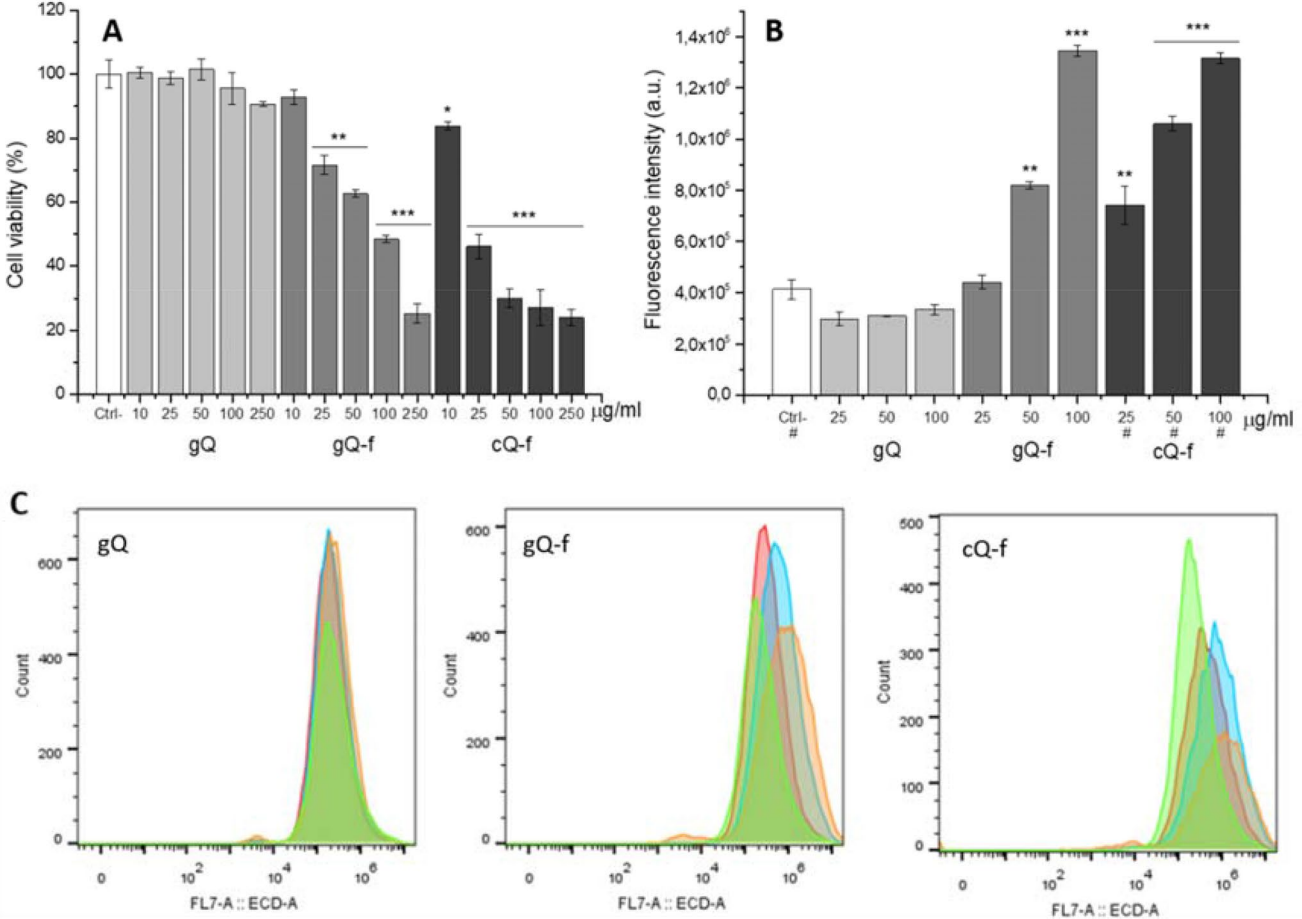
Impact of quartz particles on cell viability (**a**) and lysosome alteration (**b**, **c**), after 24 h exposure to increasing doses of quartz particles in complete RPMI. The cell viability, investigated by MTT assay (**a**), is expressed as a percentage with respect to untreated cells. The mitochondrial activity significantly decreased only upon exposure to fractured quartz (gQ-f and cQ-f). Data are the mean (± SD) of four biological replicates of one representative experiment out of three. Impact on phagolysosomes (**b**, **c**) was measured by incubation of differentiated THP-1 macrophages with quartz, followed by staining with Lyso Tracker Red dye. Fluorescence variation was evaluated by flow cytometry: mean fluorescence (**b**) and corresponding Lyso Tracker intensity distributions (**c**). Quartz concentrations are identified by the colour code described as follows: red: 25 μg/ml; cyan: 50 μg/ml; orange: 100 μg/ml; green: untreated cells. The Lyso Tracker Red data shown is the result of three biological replicates of one representative experiment out of three. Cell counts ranged from ca. 7600 to 20,000. Samples where less than 20,000 cells were counted are highlighted by a hash mark (#). For synthetic quartz samples (gQ and gQ-f), 20,000 cells were counted on average, for each dosage. Because of cytotoxicity, fewer cells were counted for cQ-f (25 μg/ml: 12,728 cells; 50 μg/ml: 13,575 cells: 100 μg/ml: 7680 cells). Untreated cells ranged from 16,300 to 18,500. Differences between negative control and quartz-exposed cells were evaluated with one-way ANOVA, followed by Tukey's post-hoc analysis. *p* values < 0.05 were considered statistically significant. *p < 0.05, **p < 0.01, ***p < 0.001 vs. control not exposed to quartz

The impact of quartz on macrophage metabolic activity upon 24 h exposure to increasing doses of the different quartz particles was investigated by the MTT assay in complete medium. In addition, since particle uptake is usually followed by the trafficking of the internalized materials to the lysosomes (Rejman et al. 2004; Nel et al. 2009; Salvati et al. 2011; Wang et al. 2013; Sabella et al. 2014), we evaluated whether alterations at phagolysosomal level could be detected. The acidic compartment of macrophages exposed to quartz was stained with the acidotropic probe Lyso Tracker Red (Fig. 3) (Wang et al. 2013). Lyso Tracker staining is often used to detect lysosomal alterations (Wang et al. 2013, 2018). An increase in its intensity upon treatment is a sign of an increase in acidity of the lysosomal compartment or increase in number or volume of lysosomes. Loss of Lyso Tracker staining can instead be a sign of lysosomal membrane permeabilization or a consequence of cell death. Cells exposed to as-grown crystals (gQ) did not show any significant metabolic or phagolysosomal alteration, even at the highest dose (Fig. 3). On the contrary, exposure to fractured synthetic crystals (gQ-f) induced a considerable reduction of cell metabolic activity and a strong increase in Lyso Tracker intensity (Fig. 3a, b). In particular, the impact on phagolysosomes was appreciable already at low particle dosage, with a clear dose–response trend and effects similar to what observed with cQ-f quartz. The flow cytometry analysis also revealed a cellular sub-population with loss of Lyso Tracker staining at higher doses (Fig. 3c, orange curves, corresponding to particle concentration of 100 μg/ml), in particular in the case of cells treated with fractured quartz dusts (gQ-f and cQ-f), possibly due to dying cells. This is largely consistent with the cytotoxicity observed in the same conditions (Fig. 3a). Overall, these results clearly indicated that fractured quartz dusts induced a decrease in cell metabolic activity and strong alterations of the acidic compartment of THP-1 macrophages.

### Fractured quartz cytotoxicity is quenched when phagocytosis is prevented

**Fig. 4:**
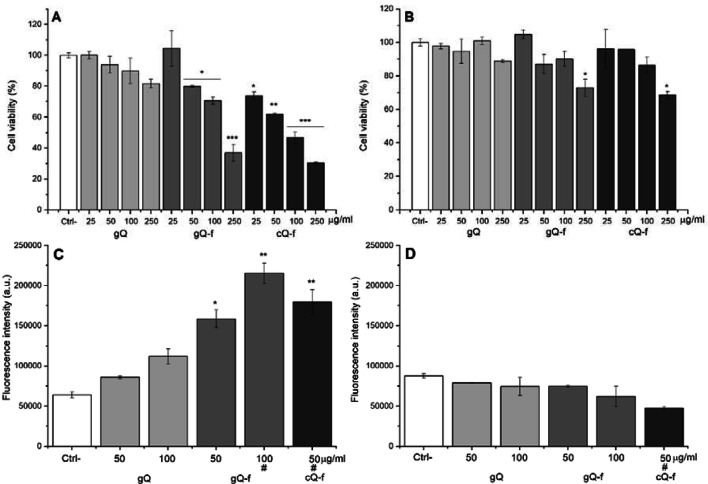
PMA-differentiated THP-1 macrophages were exposed for 4 h to increasing doses of quartz particles in cRPMI, in standard (**a**, **c**) or energy depleted (**b**, **d**) conditions, followed by particle removal and further incubation at 37 °C for 20 h. Impact on cell viability was evaluated via MTT (**a**, **b**). Data are from one representative experiment, using four replicates per dose, and are expressed as a percentage with respect to untreated cells. Phagolysosome alteration (**c**, **d**) was investigated by staining cells with Lyso Tracker Red dye, and flow cytometry. Data are the mean (± SD) of one representative experiment out of three, with two replicates for each condition. Counted cells ranged from ca. 15,000 to 20,000. Samples where less than 20,000 cells were counted are highlighted by a hash mark (#). Differences between negative control and quartz-exposed cells were evaluated with one-way ANOVA, followed by Tukey's post-hoc analysis. *p* values < 0.05 were considered statistically significant. *p < 0.05, **p < 0.01, ***p < 0.001 vs. control not exposed to quartz

To investigate whether cytotoxicity was induced via interactions at the outer cell membrane level or following particle internalization, the MTT and Lyso Tracker Red assays were repeated in energy depleted conditions to suppress active uptake processes (Rejman et al. 2004). Cells were exposed for 4 h to quartz in standard (37 °C / 4 h) or energy-depleted (4 °C / 4 h) conditions, then washed to remove extracellular particles, and incubated at 37 °C for 20 h, prior to MTT assay and Lyso Tracker staining (Fig. 4).

The cytotoxicity and phagolysosome alterations resulting after 37 °C/4 h + 37 °C/20 h exposure (Fig. 4a, c) were similar to what observed after the conventional incubation (37° C/24 h exposure, Fig. 3). On the contrary, cytotoxicity and phagolysosome alterations were almost completely suppressed when uptake was impaired (4 °C/4 h + 37 °C/20 h) (Fig. 4b, d), indicating that both cellular responses are induced by fractured quartz only upon active internalization by THP-1 macrophages.

### Hemolytic activity but not cytotoxicity is suppressed when the surface of fractured quartz is coated with FBS proteins

**Fig. 5:**
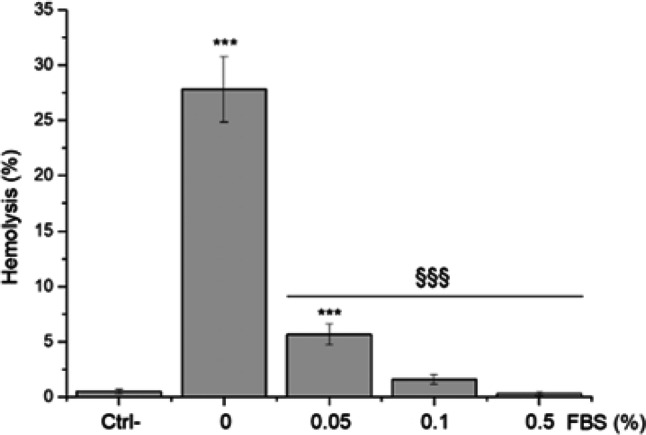
Hemolytic activity of gQ-f in absence or presence of FBS. Quartz was incubated 30 min at 25 °C with purified human red blood cells, in the presence of increasing concentrations of FBS (0, 0.05, 0.1 and 0.5%). FBS was not removed during RBCs exposure to particles. Dispersion in medium with protein reduced the hemolytic activity of quartz particles. Data were analysed with one-way ANOVA, followed by Tukey's post-hoc analysis. *p* values < 0.05 were considered statistically significant. *p < 0.05, **p < 0.01, ***p < 0.001 vs. control not exposed to quartz. §*p* < 0.05, §§*p* < 0.01, §§§*p* < 0.001 vs. hemolysis with no FBS (0% FBS)

To gain insights on whether the interaction of quartz particles with membranes is modulated by the presence of biomolecules, we compared the effect of quartz dust in the presence and absence of FBS, as a source of proteins. When quartz dusts come into contact with the lung lining fluid, the formation of a biomolecule layer, in particular proteins, possibly covering the surface of quartz is expected. To gain evidence of the formation of a protein-coating, the surface charge of quartz dust incubated with FBS was assessed. The shift in zeta-potential of ca. − 10 mV (see Table 1) confirms the formation of a protein corona on FBS-incubated quartz. The capacity of the fractured synthetic sample (gQ-f) to induce hemolysis was assessed by exposing RBCs to particles in the presence of increasing concentrations of FBS (Fig. 5).

**Fig. 6:**
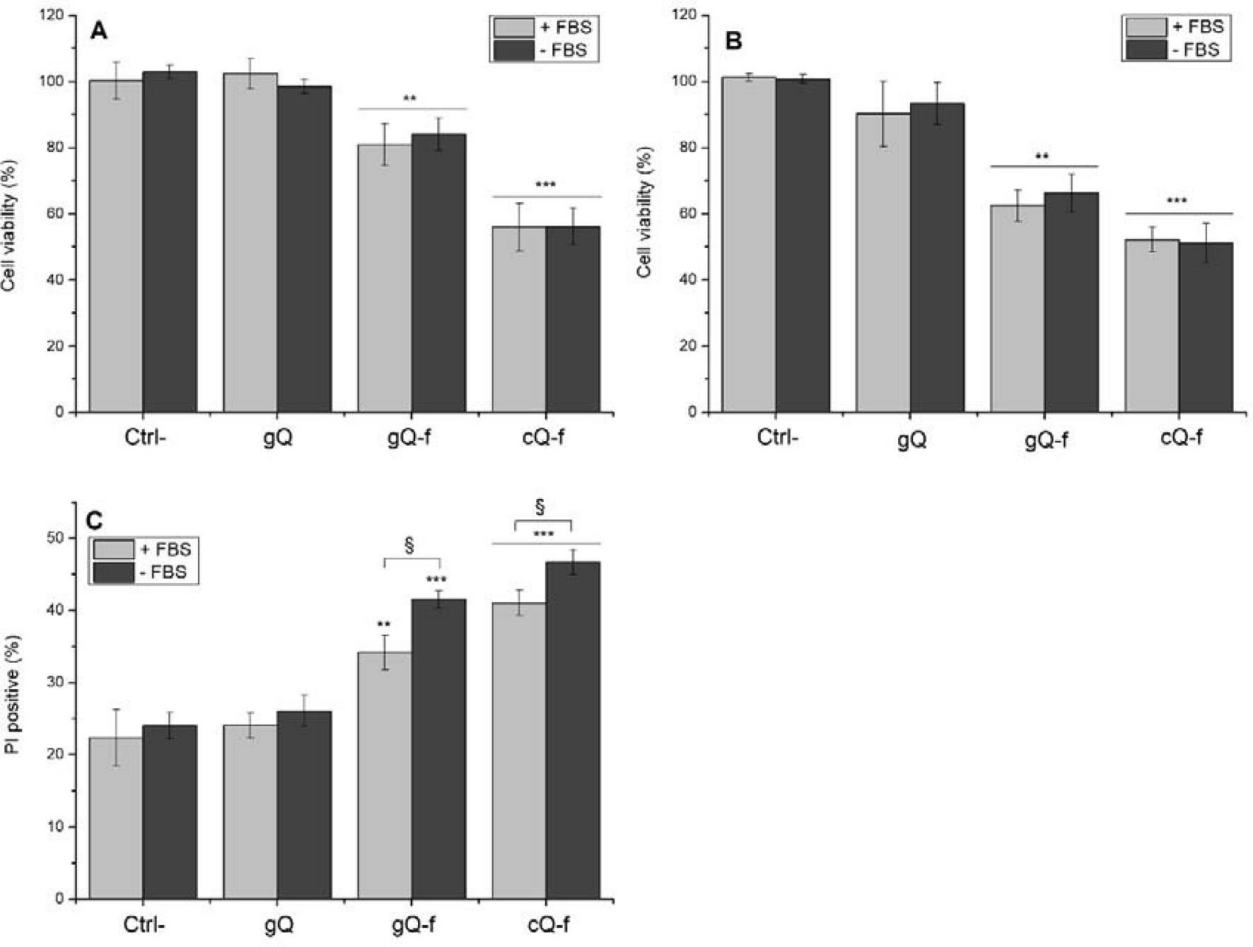
Impact of FBS on quartz cytotoxicity was evaluated via MTT assay after (**a**) 4 h and (**b**) 24 h exposure, and (**c**) % of PI-positive cells, as measured by staining with propidium iodide and flow cytometry (4 h exposure). THP-1 macrophages were exposed to particles (100 μg/ml) in presence of 10% FBS (+ FBS) or in serum-free RPMI (- FBS). Cytotoxicity is observed for fractured samples only, as expected. Comparing + FBS and—FBS exposures, MTT readouts at both 4 (**a**) and 24 h (**b**) were similar, while PI assay (**c**) showed small differences for fractured particles only. MTT data are from one representative experiment out of three, using four replicate wells per concentration, and are expressed as a percentage with respect to the values of untreated cells. PI assay data result from one representative experiment out of three, using four replicates. A variable number of cells ranging from a minimum of ca. 7000 (cQ-f) to a maximum of ca. 12,000 (gQ) were counted. Data are expressed as means (± SD). Differences between negative control and quartz exposed-cells and + FBS vs.—FBS were evaluated with one-way ANOVA, followed by Tukey's post-hoc analysis. *p* values < 0.05 were considered statistically significant. *p < 0.05, **p < 0.01, ***p < 0.001 vs. control not exposed to quartz. §*p* < 0.05, §§*p* < 0.01, §§§ *p* < 0.001 for + FBS vs.—FBS

Importantly, FBS markedly reduced the hemolytic activity of fractured crystals. This effect was observed already at the lowest concentration of FBS and attained a complete suppression of the hemolytic activity at 0.3%. To investigate the role of particle surface in quartz cytotoxicity, we exposed THP-1 macrophages to the quartz samples in presence of 10% FBS in the medium (+ FBS) or in serum-free medium (- FBS). Cytotoxicity was assessed after 4 and 24 h by MTT assay (Fig. 6a, b), and after 4 h by PI assay (Fig. 6c), used here as an additional marker of cytotoxicity. The overall cytotoxicity, as expected, increased upon the incubation with fractured crystals. The cellular metabolic activity of the cells, assessed by MTT assay, significantly decreased after 4 and 24 h incubation time. Interestingly, at both exposure times (4 and 24 h), a similar cell viability was observed when cells were exposed to fractured quartz dusts in medium with proteins or serum-free medium. The PI assay further confirmed that only fractured quartz is cytotoxic, and allowed us to evidence a small but significant difference in % of PI-positive cells when macrophages were exposed in + FBS or—FBS media.

## Discussion

The permeabilization of the lysosomal membrane can lead to the intracellular inflammasome activation and release of pro-inflammatory mediators (IL-8, IL-1β) (Mossman and Churg 1998; Latz et al. 2013; Joshi et al. 2015; Rabolli et al. 2015; Sharma and Kanneganti 2016; Hindman and Ma 2019). These events are observed upon prolonged exposure to respirable crystalline silica (RCS) and are held responsible for the induction of the persistent lung inflammation resulting, in the long term, in severe pathological outcomes, including lung cancer (Albrecht et al. 2007).

Recent works investigating the adverse outcome pathway (AOP) for quartz-induced lung pathologies (Pavan and Fubini 2017; Pavan et al. 2019) proposed the interaction of quartz with the phagolysosomal membrane as one of the key events in quartz toxicity. In this work, we investigate the effects of quartz dusts on THP-1 monocytes PMA-differentiated into macrophages, chosen as a model for alveolar macrophages, and we focus on quartz impact on the phagolysosomal membrane. To understand how quartz initiates the inflammatory process in macrophages, we measured the membranolytic activity, the cytotoxicity, and the impact on phagolysosomes elicited by the as-grown and fractured quartz crystals. The modulation of these readouts by serum proteins adsorbed on the surface of quartz was also determined and allowed to propose an uptake-dependent mechanism of action for quartz in macrophages.

Quartz crystals with fractured surfaces, both synthetic and industrially produced, show a higher membranolytic activity than as-grown quartz dust, suggesting that the former might be able to induce a stronger destabilization of the macrophage membranes. Our data also indicate that all quartz dusts are internalized by THP-1 cell, but only quartz with fractured surfaces affects cell viability and induce lysosome alterations and cell death. Such a different behaviour of rather similar specimens can be explained with the differences in surface chemistry between as-grown and fractured crystals, specifically due to the random distribution of surface functionalities (silanols and siloxanes) induced by mechanical fracturing (Turci et al. 2016; Pavan and Fubini 2017). Interestingly, the impairment of active uptake obtained with the pre-incubation of cell and quartz at 4 °C, suppresses the cytotoxic effects observed for fractured samples, including lysosomal alterations. The reduction of cytotoxicity in energy-depleted cells suggests that the toxic activity of quartz is elicited after internalization by macrophages, confirming a direct action of the fractured quartz particle surface on the phagolysosomal membrane, rather than on the outer cell membrane. A further proof that the detrimental quartz-membrane interactions take place primarily at a phagolysosomal level is given by the results obtained upon incubation of quartz with serum proteins (FBS). The suppression of the membranolytic activity when fractured quartz and RBCs are contacted in an FBS medium supports the hypothesis that serum proteins mask the surface reactive groups responsible for quartz membranolytic activity. This is in agreement with previous results (Cassel et al. 2008; Hornung et al. 2008; Pavan et al. 2014) and supports the masking effect observed with quartz coated with organic polymers (Bernstein et al. 2005; Peeters et al. 2014; Ziemann et al. 2017). On the contrary, the protein coating on fractured quartz did not modify the cell viability impact towards THP-1 macrophages at 4 and 24 h (Fig. 4). The similar cytotoxicity which we observed for uncoated and protein-coated quartz suggests that a rapid removal of the protein coating occurs after particle trafficking in the lysosomes, where the lysosomal acidity and lytic enzymes can restore the pristine fractured surfaces of quartz and, in this way, its membranolytic activity. This is also supported by the results of the PI assay, in which the slightly lower cytotoxicity of protein-coated fractured particles, in comparison to what observed in serum-free media, suggests for an initial cleaning phase of quartz surfaces inside the phagolysosome, not yet completed at the shorter exposure time. The low surface area of quartz (ca. 5 m^2^/g) accounts for this relatively rapid, yet biochemically similar, process in comparison to what has been previously evidenced for some nanoparticles accumulating in the phagolysosomes (Wang et al. 2013).

In some cases, the suppression of toxicity was observed upon protein corona formation also on amorphous silica nanoparticles (Lesniak et al. 2012; Tenzer et al. 2013; Leibe et al. 2019), but not all studies have confirmed this finding (Hsiao et al. 2019; Deville et al. 2020). The partial similarities between amorphous and crystalline silica may suggest the occurrence of common surface features on fractured quartz crystals and some amorphous silicas. In fact, it is known that the mechanical fracturing of quartz crystals may induce a partial amorphization of the surface (Nagelschmidt et al. 1952) with the formation of the so-called Beilby layer. This layer is formed when the surface reconstruction that immediately follows fracturing of SiO_2_ crystals takes place and is characterized by a pronounced structural and chemical heterogeneity of surface moieties, including silanols. The comparison of as-grown and fractured quartz surfaces evidenced that several parameters describing surface silanols, including the zeta potential and surface reactivity, significantly differed among the two solids (Turci et al. 2016). The different behaviour was explained by the chemical disorganization in the long-range spatial order of silanols introduced with fracturing. The interaction of the disorganized silanols with the lysosome membrane may represent the molecular initiating event (MIE) that triggers quartz cytotoxicity, as recently proposed by Pavan and Fubini (2017). These more heterogenous surfaces may be structurally similar to those of some amorphous silicas (Murashov et al. 2006; Rimola et al. 2013) and their occurrence may explain the cytotoxicity elicited by some amorphous silica particles after uptake and accumulation in the lysosomes (Hsiao et al. 2019; Deville et al. 2020). It would be interesting to study more in detail whether the different surface chemistry induced on quartz by fracturing is qualitatively comparable with the silanol pattern that occurs on the amorphous silica nanoparticle that shows cytotoxic effect.

Overall, our data confirm that: (1) quartz with fractured surfaces induces cytotoxicity and phagolysosomal alterations in human macrophages; (2) the quartz cytotoxic activity on THP-1 macrophages is dependent on particle uptake; and (3) the cytotoxic activity of quartz relies on direct interactions of fractured quartz surfaces with the phagolysosomal membrane.

The loss of long-range order surface moieties (silanols and siloxanes) induced by the fracturing of regular crystals, macroscopically evidenced by the appearance of the typical conchoidal fractures of quartz (Murashov and Demchuk 2005), is here confirmed to be a key physico-chemical parameter in the toxicity of crystalline silica. In addition, we have gained new insights on the molecular initiating event of quartz inflammatory mechanisms (Pavan and Fubini 2017). Indeed, we observed that the detrimental effect of quartz is induced only when quartz particles are internalized, possibly when the phagolysosomal membrane is directly interacting with fractured quartz surfaces.

The molecular mechanisms leading to the observed alterations remain to be investigated. However, the results presented here support the hypothesis that quartz particles, after being internalized and cleaned up from adsorbed extracellular molecules in the phagolysosomes, may induce lysosomal membrane permeabilization (Hornung et al. 2008). Since the activation of NALP-3 inflammasome by quartz particles requires lysosome membrane damage (He et al. 2016), our results suggest that the surface chemistry of quartz crystals may directly determine membrane destabilization, starting the well-described inflammatory process related to quartz pathological outcomes. The results obtained with this work confirm the improved model mechanism for quartz toxicity on human macrophages (Pavan et al. 2014; Turci et al. 2016; Pavan and Fubini 2017), and advance our knowledge on the alterations induced by fractured quartz on the phagolysosomal membrane, that are responsible for the initiation of quartz inflammogenic effect (Hornung et al. 2008).

## Conclusions

One of the most robust paradigm about the origin of the toxicological response to quartz dust involves the interaction of particles internalized by macrophages with the phagolysosomal membrane as the molecular initiating event (MIE) of quartz-induced cytotoxic and inflammatory response (Pavan and Fubini 2017). In the present work, we have observed that the internalization of quartz crystals by THP-1 macrophages resulted in cytotoxic effects, including a strong impact at the lysosomal level, only when quartz is fractured. Cytotoxicity was quenched when the phagocytic activity of macrophages was prevented. In parallel, we observed that FBS suppressed the membranolytic activity, but not cytotoxicity, of fractured quartz, suggesting that quartz membrane lysis takes place in the phagolysosomes, following removal of proteins adsorbed on the particle surface. This led us to conclude that the interactions of the fractured crystal surfaces with the inner side of the phagolysosomal membrane, leading to its destabilization, are likely the cause of the observed impact on cell viability. On a broader level, this work suggests that toxic surface features of quartz could be predicted by a molecular knowledge of the surface chemistry of the dust. New investigations are currently being carried out to unveil the chemical nature of the surface features responsible for the membranolytic and cytotoxic activity of quartz (Pavan et al. 2019, Pavan et al., under review/unpublished work).

## Data Availability

The datasets generated during and/or analysed during the current study are available from the corresponding author on reasonable request.

## Abbreviations

AM: Alveolar macrophages
AOP: Adverse outcome pathway
BET: Brunauer-Emmett-Teller
cRPMI: Complete RPMI (RPMI + 10% FBS)
DLS: Dynamic light scattering
ELS: Electronic light scattering
FBS: Fetal bovine serum
FPIA: Flow particle image analyser
LMP: Lysosome membrane permeabilization
MIE: Molecular initiating event
MTT: 3-(4,5-Dimethylthiazol-2-yl)-2,5-diphenyltetrazolium bromide
PFA: Paraformaldehyde
PI: Propidium iodide
PMA: Phorbol myristyl acetate
RCS: Respirable crystalline silica
RPMI: Rosewell Park Memorial Institute 1640 Medium
SEM: Scanning electron microscopy
SSA: Specific surface area
TEM: Transmission electron microscopy
